# Blockade of chemokine-induced signalling inhibits CCR5-dependent HIV infection *in vitro *without blocking gp120/CCR5 interaction

**DOI:** 10.1186/1742-4690-2-23

**Published:** 2005-04-04

**Authors:** David J Grainger, Andrew ML Lever

**Affiliations:** 1 Department of Medicine, University of Cambridge, Box 157, Addenbrooke's Hospital, Hills Road, Cambridge, CB2 2QQ, UK

**Keywords:** Chemokines, Coreceptors, seven transmemberane receptors

## Abstract

**Background:**

Cellular infection with human immunodeficiency virus (HIV) both *in vitro *and *in vivo *requires a member of the chemokine receptor family to act as a co-receptor for viral entry. However, it is presently unclear to what extent the interaction of HIV proteins with chemokine receptors generates intracellular signals that are important for productive infection.

**Results:**

In this study we have used a recently described family of chemokine inhibitors, termed BSCIs, which specifically block chemokine-induced chemotaxis without affecting chemokine ligands binding to their receptors. The BSCI termed Peptide 3 strongly inhibited CCR5 mediated HIV infection of THP-1 cells (83 ± 7% inhibition assayed by immunofluoresence staining), but had no effect on gp120 binding to CCR5. Peptide 3 did not affect CXCR4-dependent infection of Jurkat T cells.

**Conclusion:**

These observations suggest that, in some cases, intracellular signals generated by the chemokine coreceptor may be required for a productive HIV infection.

## Background

Human immunodeficiency virus (HIV) enters target cells by forming a ternary complex between the viral envelope protein gp120 and two cellular receptor proteins: CD4 and a chemokine receptor [[1-6], reviewed in [7]]. HIV viral strains have been described which use a wide range of different chemokine receptors, although the majority use either CCR5 (R5 strains), CXCR4 (X4 strains) or both of these receptors. Consistent with a requirement for chemokine receptors as cofactors for viral entry, the chemokine ligands have been reported to reduce HIV infectivity *in vitro *[8-10]. Furthermore, mutations in the gene encoding CCR5, such as the CCR5-Δ 32 allele, provide some protection against HIV infection *in vivo *[11-13]. Consequently, agents which block HIV interaction with chemokine receptors are candidate antiviral therapies which can be used in conjunction with protease inhibitors and reverse transcriptase inhibitors to attenuate a third phase of the virus life-cycle: cell entry [7,10,14,15], in the same way as the novel fusion inhibitor enfuvrtide [16]

Interestingly, the HIV gp120 protein which interacts with the chemokine co-receptor primarily through its V3 loop can induce leukocyte chemotaxis, demonstrating that some intracellular signals are generated through the the virus:receptor interaction [17,18]. This signalling occurs even though the site of the gp120 interaction with the chemokine receptors appears to be only partially overlapping with the natural ligand binding site [14,19-22].

It has been proposed that this chemotactic signalling might play a role during HIV infection *in vivo*, possibly by recruiting susceptible T-cells to sites of viral replication [18]. In other retroviruses envelope/receptor interactions are known to be mitogenic [23] and this may facilitate nuclear translocation and integration of the provirus. In HIV, however, it is not known whether the ability to productively engage the chemokine receptors in this way plays any direct role in acute viral entry and subsequent productive infection of the target cell. Guntermann and colleagues showed that pertussis toxin (which blocks G_i_-mediated signalling through chemokine receptors) block cellular infection with HIV *in vitro *[24]. Montes *et al*. obtained similar results, and also showed that the MEK inhibitor U0126 could block both chemokine-receptor-induced ERK activity and HIV infection *in vitro *[25]. However, neither pertussis toxin nor MEK inhibition are specific for chemokine signalling pathways: G_i _and ERKs participate in other intracellular signalling pathways, so it is possible that HIV infection was inhibited because of blockade of downstream pathways not initiated through productive occupancy of the chemokine receptors.

Recently, we have described a new class of chemokine inhibitors, termed Broad Spectrum Chemokine Inhibitors (BSCIs) which block chemokine-induced chemotaxis in a range of leukocytes, irrespective of the chemokine used [26,27]. These BSCIs are highly selective for chemokines, however, and have no effect on chemotaxis induced by a range of other chemoattractants such as TGF-β, fMLP or C5a. Importantly, the molecular target of the BSCIs is not the chemokine receptors themselves: BSCIs do not bind to chemokine receptors, do not affect chemokine receptor levels on the cell surface, and do not interefere with the binding of chemokine ligands to the receptors [27]. Instead, they are thought to specifically inhibit intracellular signals required for chemokine-induced migration but not for migration induced by non-chemokine pathways [27], although their molecular target has not yet been published. As a result, members of the BSCI family have been shown to be potentially useful new anti-inflammatory agents in a wide range of diseases [27].

BSCIs provide an ideal tool to probe the importance of chemokine-induced intracellular signalling in HIV infection. Since the effects of these compounds are apparently selective for chemokine receptor-induced signals, if BSCIs interefere with cellular infection by HIV *in vitro *this will indicate that productive signalling by the chemokine co-receptor is likely to be important for successful infection. In the present study, we have investigated whether the first BSCI to be described, termed Peptide 3 [26], affects gp120 binding to chemokine receptors or cellular infection by HIV *in vitro*.

## Results

### Effect of Peptide 3 on gp120 binding

**Figure 1:**
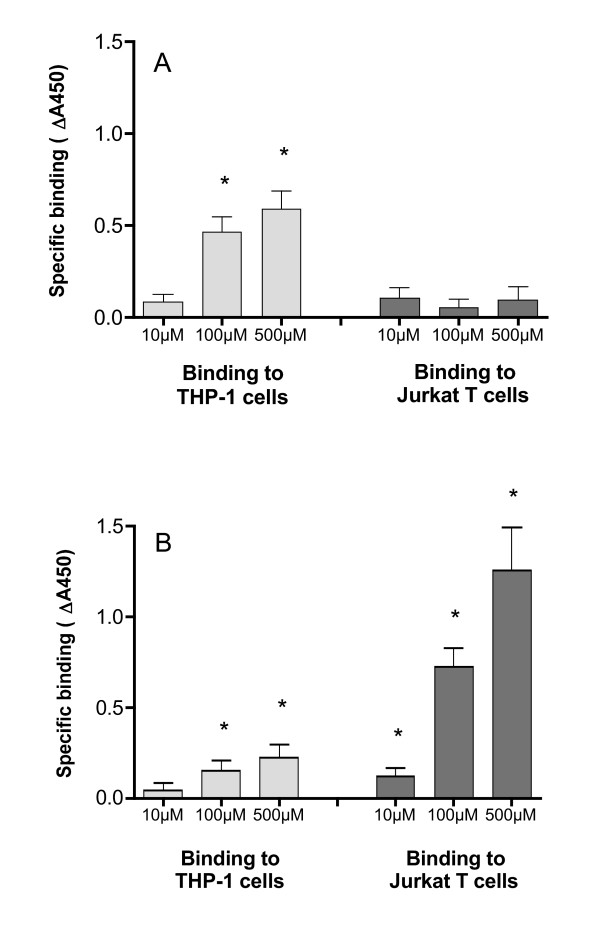
**Specific binding of gp120:V3 loop peptides to THP-1 and Jurkat cells**. (a) Binding of gp120:V3(BaL)-biotin 10^6 ^Jurkat cells or THP-1 cells per reaction were incubated with various concentrations of N-terminal biotinylated peptide in the presence and absence of 10 mM unlabelled peptide. Specific binding is expressed as the absorbance in the absence of unlabelled peptide minus the absorbance in the presence of competitor (b) Binding of gp120:V3(IIIb)-biotin under the same conditions as in (a). All reactions were performed in 100 μl of binding medium at 4°C (see Materials and Methods). Values are mean ± SEM from triplicate determinations. * p < 0.05 Student's t-test for specific binding.

The binding of gp120 to chemokine receptors is likely to involve sequences in the V3 loop of gp120 [28,20]. We therefore synthesised peptide sequences from the V3 loop of the M-tropic BaL strain and the T-tropic IIIb strain and analysed the binding of these biotinylated peptides to the THP-1 and Jurkat cells. Specific (competable) binding of gp120:V3(BaL) to THP-1 cells was detected at 100 μM (Fig 1a). In contrast, specific binding of gp120:V3(BaL) to Jurkat cells was not detected even at concentrations up to 500 μM (Fig 1a). These observations are consistent with the hypothesis that gp120:V3(BaL) binds specifically to CCR5, which is expressed on the surface of THP-1 monocytic cells but not on Jurkat T-cells.

Specific binding of gp120:V3(IIIb) at 100 μM to both Jurkat T-cells and THP-1 cells was detected. There was approximately 5-fold greater specific binding to the Jurkat cells than the THP-1 cells (Fig 1b). These observations are consistent with the hypothesis that gp120:V3(IIIb) binds specifically to CXCR4, which is expressed on both THP-1 and Jurkat cells, but at higher levels on the T-cell line.

**Figure 2:**
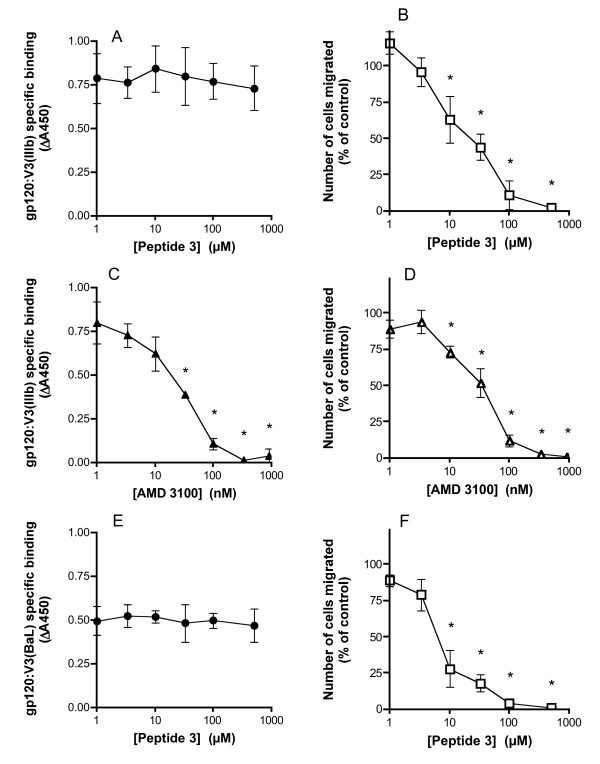
**Effect of Peptide 3 on gp120:V3 loop peptide binding to cells**. (a) The binding of 100 μM N-terminal biotinylated gp120:V3(IIIb) to THP-1 cells was measured in the presence of various concentrations of peptide 3. In each case, the non-specific binding (in the presence of 10 mM unlabelled gp120:V3 loop peptide) has been subtracted. (b) Chemotaxis in response to 100 ng/ml SDF1α was measured in the presence of various concentrations of peptide 3. (c) and (d) As for (a) and (b) except that the CXCR4 receptor antagonist AMD3100 was used in place of Peptide 3. (e) As for (a) except that the effect of Peptide 3 on the binding of gp120:V3(BaL) to THP-1 cells was determined. (f) As for (b) except that the effect of Peptide 3 on chemotaxis induced by 25 ng/ml MIP1α was determined. All binding reactions were performed with 10^6 ^cells in 100 μl of binding medium at 4°C. Chemotaxis assays were performed with 5 × 10^4 ^cells per well. Values are mean ± SEM of triplicate determinations.

We next incubated THP-1 and Jurkat cells with 100 μM of each biotinylated gp120:V3 peptide in the presence of various concentrations of Peptide 3. Peptide 3 had no effect on the binding of gp120:V3(IIIb) to Jurkat cells (Fig 2a), even though it powerfully inhibited SDF-1α induced chemotaxis over the same concentration range (Fig 2b). Under the same conditions, the CXCR4 receptor antagonist AMD3100 [31] blocked both gp120:V3(IIIb) binding and SDF-1α-induced migration with similar IC50s (Fig 2c, d). Similarly, Peptide 3 had no effect on the binding of gp120:V3(BaL) to THP-1 cells (Fig 2e).under conditions where RANTES-induced chemotaxis was powerfully inhibited (Fig 2f). Taken together, these data confirm that Peptide 3 blocks chemokine signalling without blocking gp120 interaction with the chemokine receptors, consistent with previous observations that BSCIs such as Peptide 3 do not block chemokine ligand interactions with their receptors [27].

### Effect of peptides on HIV infection in vitro

**Figure 3:**
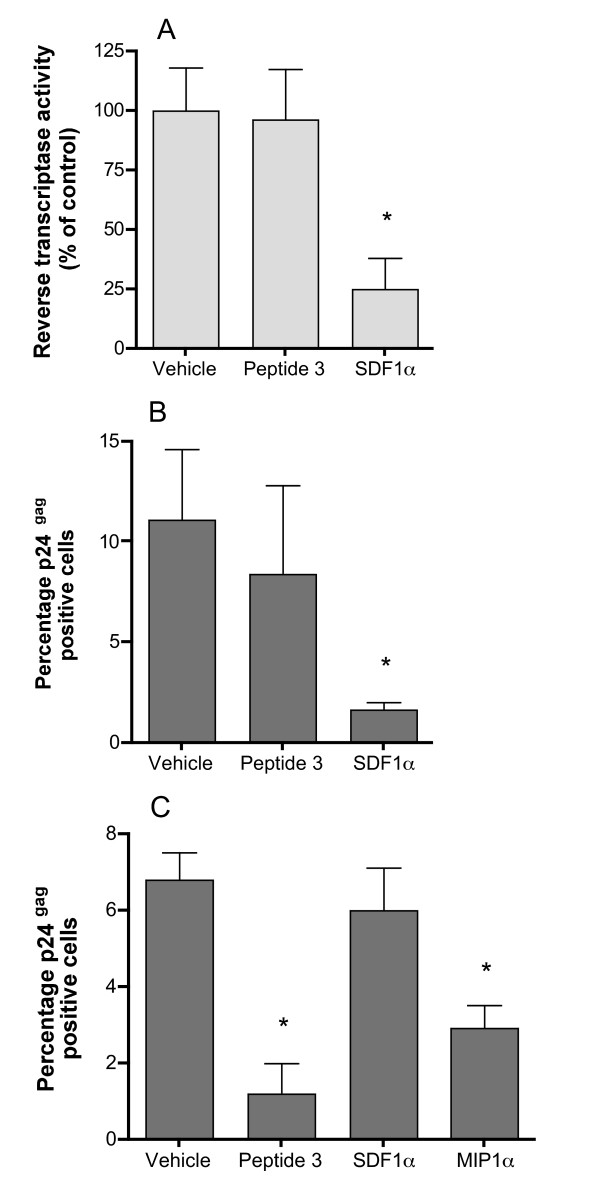
**Peptide 3 inhibition of HIV infectivity *in vitro***. (a) HIV (IIIb) replication in cultures of Jurkat T-cells was estimated by measuring the supernatant reverse transcriptase activity two weeks after infection. Peptide 3 was at 100 μM final concentration and SDF-1α was added at 100 ng/ml final concentration 1 hour prior to exposure to virus. Values are mean ± SEM from 12 wells, expressed as the percentage of the reverse transcriptase activity in the supernatant from the control wells. The experiment shown is typical of six separate experiments. (b) HIV (IIIb) infectivity of Jurkat T-cells was estimated by staining cells treated identically to those in (a) for p24^*gag *^expression. Values are mean ± S.D. percentage of cells stained for p24^*gag *^averaged from 12 fields of view from each of two separate wells. (c) HIV (MN) infectivity of THP-1 cells measured as in (b). MIP1α and SDF1α were used at 100 ng/ml final concentration.

HIV infection of Jurkat T-cells using the laboratory-adapted T-tropic isolate IIIb was monitored using two different assays. Firstly, Jurkat T-cells in 96-well plates were pre-treated with either Peptide 3, vehicle (as a negative control) or SDF1α (as a positive control) for 4 hours, then exposed to HIV virus (10^6 ^TCID_50_) and pulsed at 2–3 day intervals with Peptide 3, SDF1α or medium alone as appropriate. After two weeks in culture, the extent of viral infection was assayed by measuring the reverse transcriptase activity in the supernatant, as a measure of viral replication in the culture [32] In six separate experiments, Peptide 3 (100 μM) had no effect on virus replication following HIV exposure (Fig 3a), while SDF1α inhibited reverse transcriptase activity by an average of 75%. No effect was seen on cell viability under any of the treatment conditions.

HIV infection of Jurkat T-cells was also monitored by high sensitivity quantitative immunofluoresence detection of viral p24^gag ^expression. Jurkat cells were infected with HIV in the presence or absence of Peptide 3 (100 μM) or SDF1α (100 ng/ml) as described above. Approximately 48 h after infection, the cells were attached to glass slides using a cytospin and then fixed by immersion in ice-cold 70% ethanol for 90 seconds. Expression of p24^gag ^was determined using quantitative immunofluoresence as previously described [33], except that the primary antibody was post-fixed to the section using paraformaldehyde to increase the sensitivity of the technique (see Methods). Viral infectivity was expressed as the number of cells stained for p24^gag ^expressed as a proportion of the total number of cells (detected using Hoechst 33342 nuclear dye). Consistent with the reverse transcriptase assay results, SDF1α inhibited viral infectivity by more than 80% (Fig 3b), while peptide 3 had no effect.

Infection of THP-1 cells with M-tropic isolates does not generate high levels of virus particles and hence the reverse transcriptase assay is not sufficiently sensitive to monitor the progress of the infection. However, it was possible to assess HIV infectivity of THP-1 cells using high sensitivity immunofluorescent detection of p24^gag^. THP-1 cells were differentiated with hydrocortisone and PMA, then treated with TNFα, resulting in adherent monolayers on glass chamber slides. The THP-1 cells were then treated with either Peptide 3 (100 μM), MIP1α (100 ng/ml) or SDF1α (100 ng/ml) as for the Jurkat cells. THP-1 cells were infected with HIV strain MN at a concentration previously validated to produce easily detectable infection and grown for 72 h prior to fixation and staining for p24^gag^. In contrast to the findings with HIV strain IIIb infection of Jurkat T cells, Peptide 3 inhibited infection of THP-1 cells by more than 80% (Fig 3c), very similar to the effect of MIP1α. SDF-1a had no statistically significant effect on HIV strain MN infection of THP-1 cells, confirming that the infection was entirely CCR5-dependent, even though THP-1 cells express CXCR4.

## Discussion

Taken together our results suggest that, at least under some conditions, the generation of intracellular signals by the chemokine co-receptor during HIV infection might be necessary for productive infection. Since Peptide 3 powerfully inhibited CCR5-dependent HIV infection of THP-1 cells under conditions where gp120 binding to CCR5 was unaffected but chemotaixs in response to RANTES was profoundly blocked, it is likely that at least some of the signals elicted by CCR5 occupation that result in chemotaxis are required for successful infection of the cell by HIV. Since BSCIs, such as Peptide 3, do not block chemokine receptor internalisation induced by ligand binding [27], it seems likely that the HIV successfully entered the cell in the presence of Peptide 3, but that some later stage in the viral life cycle was dependent on one or more intracellular signal generated by chemokine receptor occupancy. These results are consistent with, but extend, the findings of Guntermann [24] and Montes [25] who saw similar effects with pertussis toxin and a MEK inhibitor.

It is unclear why Peptide 3 blocked CCR5-dependent HIV infection of THP-1 cells but had no effect on CXCR4-dependent infection of Jurkat T-cells under similar condtions (even though Peptide 3 efficiently blocks SDF1α dependent chemotaxis). It is possible that infection of certain cell types (such as monocyte/macrophage cells) is more dependent on a chemokine receptor-induced intracellular signal than infection of other cell types (such as T-lymphocytes). This may reflect the fact the the Jurkat cells were proliferating at the time of infection, whereas the THP-1 derived macrophages were quiescent. However, it is also possible that this difference is due to the particularly high levels of CXCR4 which are expressed on Jurkat T cells. The high levels of receptor on this cell line might render infection relatively insensitive to intracellular signalling requirements compared with native T-cells or other cell types expressing physiological levels of chemokine co-receptors.

Irrespective of the reasons for this difference, our preliminary studies illustrate the need to further investigate the role of intracellular signals induced by co-receptor occupancy as participants in the viral life cycle. Furthermore, the recent discovery of much more potent non-peptide BSCIs, such as the acylaminocaprolactams [34,35], opens up the possibility that interfering with chemokine receptor-induced signalling might offer an alternative therapeutic strategy to blocking chemokine receptor binding. The best acylaminocaprolactam BSCIs are cheap, orally bioavailable and apparently free of acute toxicity [35], and, on the basis of studies such as ours, warrant further investigation as part of an antiviral combination therapy for HIV.

## Conclusion

We conclude that selective inhibition of the intracellular signal(s) generated through interaction of the HIV gp120 envelope protein with its chemokine co-receptor can block productive HIV infection in THP-1 derived macrophages in vitro. However, blockade of CXCR4-mediated signalling in Jurkat T-cells has no effect on HIV infection in vitro. Under certain condtions, therefore, HIV infection may require activation of the chemokine co-receptor signalling pathway.

## Methods

### Peptides

Peptides were prepared by Affiniti (Exeter, U.K.) using standard solid phase chemistry, followed by reverse-phase HPLC purification to greater than 95% purity. Peptide 3 (derived from amino acids 51–62 of mature human MCP-1) had the sequence EICADPKQKWVQ [26]. Labelled peptides were also synthesised corresponding to the sequence of the full length V3 loop (including terminal cysteine residues) of gp120 from HIV-1 IIIb and HIV-1 BaL with an N-terminal co-synthetic biotin label. All peptides were prepared as TFA salts and dissolved in sterile MilliQ and stored at -20°C until used.

### Chemotaxis experiments

Chemotaxis experiments were performed essentially as previously described [26,36], using the ChemoTx disposable 96-well transwell migration plates, with PVP-free membrane (6 μm pore size). Chemoattractant (lower compartment) and cells upper compartment) were suspended in Gey's Balanced salt solution + 1 mg/ml BSA. Consistent with our previous recommendations [36], putative inhibitors were added at equal concentration to both the upper and lower compartments. Migration was allowed to proceed for 2 hours at 37°C. The number of cells which had migrated to the lower compartment was determined using the vital dye MTT and interpolation of a standard curve. Each condition was determined in triplicate, and the number of cells migrating in the absence of chemoattractant was subtracted to determine the chemokine-dependent chemotaxis which was reported.

### Binding assays

Cells (either Jurkat T-cells or THP-1 cells) were grown in RPMI 1640 medium supplemented with 10% fetal calf serum, 2 mM glutamine, 20 μM β-mercaptoethanol, 100 U/ml penicillin and 100 μg/ml streptomycin and maintained between 2 × 10^5 ^and 1 × 10^6 ^cells/ml. Prior to performing a binding assay, cells were spun out (100 × g; 4 mins) and washed 3 times in ice-cold PBS. A volume of cell suspension in PBS containing 10^6 ^cells was pipetted into each well of a V-bottom 96-well plate (Gibco BRL) and spun out (100 × g; 4 mins). Cells from triplicate wells were then resuspended in 100 μl binding medium (PBS pH 7.2 containing 0.1% fatty-acid free bovine serum albumin (BSA)) containing labelled peptide in the presence or absence of 10 mM unlabelled peptide. The plate was then incubated on ice for 90 minutes. Cells were washed 3 times with 380 μl of ice-cold PBS, spinning out the cells each time (100 × g; 4 mins), and resuspended in 100 μl binding medium containing streptavidin-peroxidase (Amersham International) at 1:1000 dilution. Cells were incubated for a further 15 minutes on ice to allow labelling of any bound biotinylated peptide, then washed 4 times as above. Cells were finally incubated with 200 μl TMB substrate (K-Blue, Bionostics) for 20 minutes at room temperature, and the reaction stopped by addition of 50 μl 2 M HCl. The plate was spun (3,000 × g ; 3 mins) and 200 μl of the coloured product was transferred to an empty 96-well ELISA plate and the absorbance at 450 nm determined.

### HIV-1 infection and reverse transcriptase assays

HIV-1 stocks were prepared in the following manner. HIV-1 IIIb (provided by the MRC AIDS Reagent Programme) was used to infect Jurkat T cells and the progression of infection was monitored using the reverse transcriptase assay for 10–14 days. Virus was then harvested and assayed for infectivity using a TCID50 assay with Jurkat T cells as targets as previously described [37]. Virus containing supernatants were centrifuged to remove cellular debris and then stored in aliqouts in liquid nitrogen until used. Stocks of HIV-1 MN strain were prepared in a similar manner, except that H9 T cells were used and the progression of infection was monitored by immunofluoresence (because of the low RT activity) in this strain.

Experimental infection of Jurkat T cells was performed by incubating cells win the presence of test peptide or chemokine with aliquots of stored virus at the titres described in the text. Thereafter, the cells were fed fresh medium containing test peptide or chemokine where appropriate, every 48 h. Viral replication two weeks after infection was estimated by measuring reverse transcriptase activity in the supernatant using the Potts Mini RT assay as previously described [32].

THP-1 cells were differentiated prior to infection with hydrocortisone and PMA in 8-well chamber slides. Sixteen hours prior to infection, TNFα was added (100 ng/ml). Twelve hours later, the medium was aspirated and replaced with fresh medium containing the test peptides or chemokines as appropriate. After a further four hours, virus from the frozen stocks was adeed to the cells, which were the processed for immunofluoresence between 28 h and 72 h after infection.

### Immunofluorescence detection of p24^gag^

Jurkat cells following HIV infection were attached to 8-well chamber slides (Becton-Dickinson) by spinning the slides using a plate rotor in a Labofuge centrifuge (Heraeus) at 3,000 × g for 5 minutes. Attached Jurkat cells or THP-1 cells were then fixed by dipping the slides into ice-cold 70% ethanol for 90 seconds. Non-specific binding was blocked by incubation with 3% fatty acid-free BSA in TBS for 1 hour at room temprature. Cells were incubated with the mouse monoclonal anti-HIV-1 p24^gag ^antibody EH12E1 (ref 38;AIDS Reagent Program, NIBSC) at 10 μg/ml in 3% BSA in TBS at room temperature overnight. Unbound antibody was removed with 3 × 3 min washes in PBS, and bound antibody was then fixed to the slide by incubation with 3.8% phosphate buffered formalin pH7.2 for 10 mins at room temperature, followed by 3 further 3 min washes in PBS. Bound antibody was visualised using donkey anti-mouse IgG FITC conjugate (715-095-150; Jackson Immunoresearch) at 30 μg/ml in 3% BSA/TBS + 1 ng/ml Hoescht 33342 for 6 hours at room temperature. Twelve fields of view (100× magnification) were captured from each well of the chamber slide using an Olympus Provis AX electronic microscope connected to a Power Macintosh 8500, running OpenLab image analysis software (Improvision), under both FITC illumination conditions (NIBA filter block; λex = 470–490 nm, dichroic mirror = 505 nm, λem = 515–550 nm) and UV illumination conditions (Chroma 31000; λex = 340–380 nm, dichroic mirror = 400 nm, λem = 435–485 nm). Images were acquired with a Hamamatsu C4742-05 monochrome digital camera with 10-bit depth in a 1280-1024 pixel field connected to a DIG Snapper frame grabber. The exposure time, amplifier gain and offset values were controlled by the OpenLab software and were held constant throughout the experiment. A background (an image captured without a slide under the objective) was digitally subtracted from every image. A threshold was then applied to each image which was the lowest threhold that detected <1% of the pixels of an image of uninfected cells stained under identical conditions. The number of objects exceeding this threshold in each field of view were counted. A similar procedure was used to determine the total number of nuclei in the same field of view, using the image captured under UV illumination conditions. The ratio of positively stained objects to nuclei in each field of view was reported as the percentage of cells stained for p24^gag^.

## Competing interests

DJG is an inventor on a range of patents filed by the University of Cambridge containing composition of matter and pharmaceutical use claims for a wide range of BSCIs, including Peptide 3 used in this manuscript. The patent specifically claims the use of BSCIs for the prevention and/or treatment of HIV infection. An exclusive license to these patents have been granted by the University of Cambridge to Ipsen (Paris, France) and DJG may gain financially from the successful exploitation of this intellectual property.

## Authors' contributions

DJG and AML jointly conceived of these studies; AML performed the HIV infection experiments and RT assays; DJG performed the immunofluoresence detection analyses and the *in vitro *binding assays and functional migration assays. DJG drafted this manuscript, which was critically reviewed by AML and both authors approve the final version for submission and publication.
